# Relationship between activity and sleep, as measured through a wearable accelerometer, and appropriate cardioverter defibrillator interventions: a prospective SafeHeart substudy

**DOI:** 10.1093/europace/euae241

**Published:** 2024-09-20

**Authors:** Diana M Frodi, Maarten Z H Kolk, Søren Z Diederichsen, Joss Langford, Reinoud E Knops, Hanno L Tan, Tariq O Andersen, Peter Karl Jacobsen, Niels Risum, Fleur V Y Tjong, Jesper Hastrup Svendsen

**Affiliations:** Department of Cardiology, Copenhagen University Hospital—Rigshospitalet, Inge Lehmanns Vej 7, 2100 Copenhagen, Denmark; Department of Clinical and Experimental Cardiology, Amsterdam UMC, University of Amsterdam, Amsterdam, The Netherlands; Department of Cardiology, Copenhagen University Hospital—Rigshospitalet, Inge Lehmanns Vej 7, 2100 Copenhagen, Denmark; Activinsights Ltd., Kimbolton, UK; College of Life and Environmental Sciences, University of Exeter, Exeter, UK; Department of Clinical and Experimental Cardiology, Amsterdam UMC, University of Amsterdam, Amsterdam, The Netherlands; Department of Clinical and Experimental Cardiology, Amsterdam UMC, University of Amsterdam, Amsterdam, The Netherlands; Netherlands Heart Institute, Utrecht, The Netherlands; Department of Computer Science, University of Copenhagen, Copenhagen, Denmark; Department of Cardiology, Copenhagen University Hospital—Rigshospitalet, Inge Lehmanns Vej 7, 2100 Copenhagen, Denmark; Department of Cardiology, Copenhagen University Hospital—Rigshospitalet, Inge Lehmanns Vej 7, 2100 Copenhagen, Denmark; Department of Clinical and Experimental Cardiology, Amsterdam UMC, University of Amsterdam, Amsterdam, The Netherlands; Department of Cardiology, Copenhagen University Hospital—Rigshospitalet, Inge Lehmanns Vej 7, 2100 Copenhagen, Denmark; Department of Clinical Medicine, Faculty of Health and Medical Sciences, University of Copenhagen, Blegdamsvej 3B, 2200 Copenhagen, Denmark

**Keywords:** Wearable technology, Wearable activity tracker, Behavioural patterns, Ventricular arrhythmia, Implantable cardioverter defibrillator, Prediction

## Abstract

**Aims:**

Physical activity has shown association with ventricular arrhythmia, however, the role of specific behavioral patterns over a 24 h cycle remains unknown. Therefore, we aimed to explore associations between physical behavior and appropriate implantable cardioverter defibrillator (ICD) therapy.

**Methods and results:**

We included patients with an ICD at two European sites, who wore wrist-based accelerometers capturing 24 h movement and sleep behaviours for 28 days. Behavioural measures included activity volume, duration and intensity, sleep duration, and efficiency. Participants were followed for 12 months for the outcome of appropriate ICD therapy. Cox proportional hazard models with restricted cubic splines were used for the analysis. Lastly, the predictive capacity was tested. A total of 253 ICD patients were included (mean age 63.5 (±10.2), 48 (19.0%) female). During follow-up, 40 participants (15.8%) received appropriate ICD therapy; 32 anti-tachycardia pacing (ATP) only (12.6%), 5 shock only (2.0%), and 3 combined ATP and shock (1.2%). In the adjusted model, high inactive duration (HR 1.40 (95% 1.10–1.78)), peak walking cadence (HR 1.07 (95% 1.03–1.12)), and total sleep duration (HR 1.50 (1.02–2.22)) were associated with the outcome. The dose–response relationship was U-shaped for inactive duration with a cut-off at 16 h, and linear for peak cadence and sleep. The prediction model reached an area under the receiver operating characteristic curve of 0.70 ± 0.03, with highest accuracy in the first months.

**Conclusion:**

Wearable-derived 24 h movement and sleep behaviours collected over 28 days were associated with later appropriate ICD therapy risk. Testing of the predictive value of digital biomarkers for enhanced risk stratification of ventricular arrhythmia warrants larger prospective studies.

**Clinical Trial Registration:**

National Trial Registration (NL9218, http://onderzoekmetmensen.nl/).

## Graphical Abstract

**Graphical Abstract:**
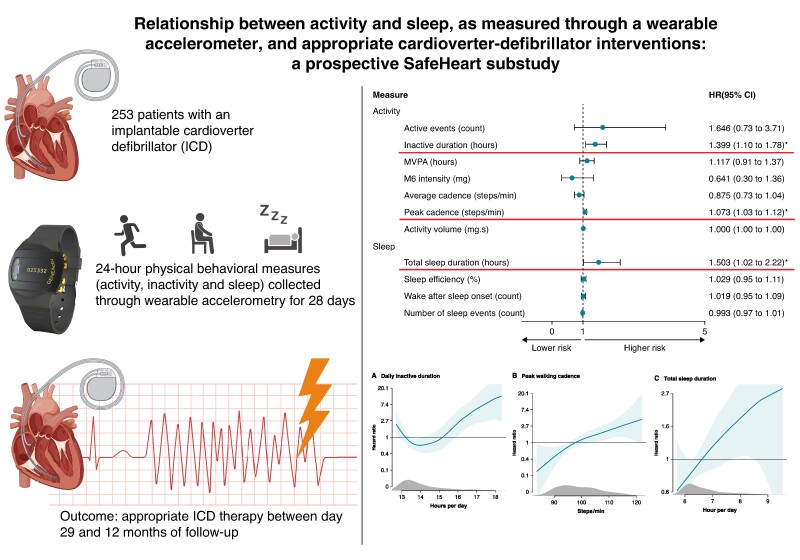

**What’s new?**

- Physical activity has shown association with ventricular arrhythmia, however, the role of specific behavioural patterns remains unknown in patients with an implantable cardioverter defibrillator (ICD).
- We measured 24 h activity and sleep behaviour over 28 days and used these measurements to explore the associations between behavioural measures and appropriate ICD therapy (shock and anti-tachycardia pacing).
- In adjusted Cox proportional hazard models, increased inactive duration (HR 1.40, 95% CI 1.10-1.78), peak walking cadence (HR 1.07, 95% CI 1.03-1.12), and total sleep duration (HR 1.50, 95% CI 1.02−2.22) were associated with the outcome.
- The dose–response curve for inactive duration was U-shaped, with a cut-off of inactivity > 16 h substantially increasing the risk of appropriate ICD therapy.
- Digital biomarkers reflecting 24 h physical behaviour collected through wrist-worn accelerometry provide additional information on the risk of appropriate ICD therapy, independent from clinical patient characteristics.

## Introduction

Implantable cardioverter-defibrillators (ICDs) are effective for prevention of sudden cardiac death, however, patients remain at risk of ventricular arrhythmias.^1^ Monitoring of activity levels has been proposed as a means to assess the risk of heart failure progression, atrial fibrillation, and sudden cardiac arrest.^2–5^ The emerging field of wearable technology has resulted in a wide availability of research- and consumer-grade wearable activity trackers that register activity types and levels in remote settings and allow for more reliable data compared to self-report.^6,7^ In addition, wearable devices can provide objective measurements across a wide spectrum of behaviours such as intensity, volume, and duration of activities, as well as sleep behaviour. Prior studies have predominantly used brief monitoring intervals of up to 7 days and often relied on daily activity level as the single measurement.^3,8^ However, these approaches fail to acknowledge the highly interrelated daily activity and sleep behaviours within a 24 h period. The effect on a given outcome might not be because of a change in a particular behaviour (activity, inactivity, or sleep), but rather a reallocation of time from one to another.^9^ These complexities require comprehensive monitoring of physical behaviour that considers the entire spectrum of activities and their relationships, thus removing the uncertainty that otherwise arises with long periods of unknown behaviour. Moreover, while previous studies have mainly used data from large biobank studies to assess associations between activity and risk of ventricular arrhythmia, there is a lack of evidence on physical behaviour in patients with an ICD.^2,3^

In this prospective study, we aimed to explore the associations between physical behaviours and the risk of appropriate ICD therapy in an international cohort of ICD patients.

## Methods

### Study design and participants

We present a substudy of the multicentre, prospective, observational SafeHeart study that was conducted at two European University Hospitals (Amsterdam University Medical Center in The Netherlands and Copenhagen University Hospital—Rigshospitalet in Copenhagen, Denmark) after approval by the Institutional Review Board and Regional Ethics Committee. The rationale and design of the study have been published previously and encompassed participants that in the past five years prior to enrolment received an ICD or in the eight years prior to enrolment experienced an appropriate or inappropriate ICD shock or/and experienced documented ventricular arrhythmia.^10^ Enrolment in the SafeHeart study took place between May 2021 and September 2022 and included patients with an ICD with or without resynchronization (CRT-D), regardless of manufacturer. The Strengthening the Reporting of Observational Studies in Epidemiology (STROBE) reporting guidelines for observational studies were followed,^11^ and the study was carried out according to the Declaration of Helsinki.

### Data collection

During the study, participants were asked to wear a passive, tri-axial accelerometer (GENEActiv Original, Activinsights Ltd., Cambridgeshire, UK) on their wrist day and night. The wearable recorded behavioural measures at 50 Hz or 20 Hz. For this particular analysis, we used the first 28 days of wearable measurements as the baseline. Participants with <18 days of valid wear days during this period were excluded from the analysis. The valid day criterium was wear of at least 22 h. The full length of follow-up in the SafeHeart study was 12 months from enrolment. As the first 28 days of follow-up were used to create the baseline, outcomes that took place from day 29 until end of study were included in this study.

### Behavioural measures and processing

Data were passively captured during wear. Once the wearable was returned to the research group, data were extracted through a USB port. Next, raw data were parsed and transformed into daily summaries of the various behavioural measures, from which behavioural time-series data could be created. The open source CRAN packages GENEAread and GENEAclassify used by the Activinsights R Markdown Sleep report were used to process the raw accelerometry-derived data.^12^ The daily behavioural measures used that were related to activity were activity volume (m*g*·s, *g*·s, units of gravity acceleration seconds), number of active events (count), M6 intensity [m*g* (*g*, units of gravity acceleration)], average and peak walking cadence (steps/day), moderate to vigorous physical activity (MVPA) (hours) and inactive duration (hours), and those related to sleep; total sleep (hours), sleep efficiency (%), wake after sleep onset events (count), and number of sleep events (count). The behavioural measures are described in detail in *Table 1* and were chosen based on their ability to consistently explain the most variance both within and between participants.

**Table 1:** Behavioural measures

Behavioural measures	Definitions
Activity	
Active event^a^ (count)	Active event number per 24 h.
Inactive duration (hours)	Total duration spent inactive [<40 m*g* average acceleration (*g*, units of gravity acceleration)] per 24 h, excluding sleep.
MVPA duration (hours)	Total duration spent in moderate- and vigorous physical activity per 24 h (>644 m*g* of acceleration magnitude, e.g. moderate paced walking or leisure activities like light bicycling).
M6 intensity (m*g*)	Intensity of the most active 6 min in 24 h (*g*, units of gravity acceleration).
Average cadence (steps/day)	The mean walking cadence (steps/minute) per 24 h.
Peak cadence (steps/day)	The 95th percentile of walking cadence per 24 h, i.e. the walking speed when walking purposefully.
Activity volume (m*g*·s)	Activity volume = sum of durations × mean acceleration for each event (*g*·s, units of gravity acceleration seconds) per 24 h.
Sleep	
Total sleep duration (hours)	Total sleep duration within rest interval.
Sleep efficiency (%)	Percentage of sleep within rest interval.
WASO count	Wake after sleep onset—number of inactive and active events (>10 s length) of non-sleep taking place during the sleep interval.
Number of sleep events (count)	Discrete sleep events and postures within rest interval.

^a^Events are durations of ≥10 s of homogeneous acceleration.

### Outcome

In this study, the outcome was appropriate ICD therapy [shock and/or anti-tachycardia pacing (ATP)]. Upon receiving ICD therapy during follow-up, intracardiac electrograms at the time of device therapy were obtained from the ICD remote monitoring systems. Adjudication of all outcomes, classified as appropriate ICD therapy (if they occurred for ventricular fibrillation or sustained ventricular tachycardia) or inappropriate ICD therapy (e.g. ICD therapy for any other rhythm and error of the device) was performed by a committee of electrophysiologists at the two study centres. Any disagreements were then solved through discussion. In this paper, the first occurring appropriate ICD therapy during the follow-up period was included for analysis. We applied a blanking period that spanned the first 28 days of participation, during which baseline activity data were collected, to limit the risk of inverse causality from ICD shocks potentially affecting activity behaviour.^13^

### Statistical analysis

Continuous variables were presented by the median, mean, interquartile range (IQR), and standard deviation. Categorical data were provided as frequency (percentages) and compared using Fisher’s exact test or χ^2^ test as appropriate. Depending on the data distribution, independent *t*-tests or the nonparametric Mann–Whitney *U* test was applied for comparisons of the mean. Associations between behavioural measures and the outcome of interest were assessed using Cox proportional hazards models and presented as hazard ratios (HRs) with 95% confidence intervals (CIs). Schoenfeld residuals were computed to test the proportional hazards assumption. For each of the behavioural measures, we built two separate Cox models. The first model included only the behavioural measure, whereas the second model was adjusted for the clinical variables age, sex, ischaemic heart disease, ICD prevention type (primary vs. secondary), device type (ICD vs. CRT-D), and heart failure diagnosis. A sensitivity analysis was performed to assess the associations between daily behavioural measures and inappropriate ICD therapy, as well as appropriate shock-only treatment. Again, the first occurring episode during follow-up was used. Additionally, we used restricted cubic splines with four knots to determine the dose–response association between physical behaviour and ICD therapy for variables significant in the adjusted Cox model. The behavioural measures that showed a significant association with the outcome in the adjusted Cox model were split into two groups based on their medians and presented in survival curves to describe their relationship to the risk of ICD therapy. Cumulative incidence of ICD therapy during follow-up was calculated using the Aalen–Johansen estimator with death as competing risk. To account for censoring and competing events, Gray’s test was used for the groupwise comparisons of absolute risks. Crude event rates were estimated with Poisson distribution and were presented as events per 100 person-years. Finally, to assess the predictive performance of the behavioural measures, we trained a multivariable Cox’s proportional hazard’s model with elastic net penalty (CoxNet). This approach combines LASSO and ridge regularization, resulting in a model that is both sparse and capable of retaining significant predictors while mitigating overfitting. This is especially useful in settings with relatively small cohorts where the number of predictors is close to the number of events. By using this machine learning technique, we were able to assess the predictive performance of the behavioural metrics alongside clinical features. We trained the model using the set of behavioural measures (outlined in *Table 2*), in conjunction with the same clinical variables used in the Cox Proportional Regression model. The model’s performance was evaluated through five-fold cross-validation. In this process, the model was trained on four of the folds not used as the validation set and tested on the remaining fold. This was repeated five times, with each fold serving as the validation set once. We evaluated the model’s performance by calculating the dynamic area under the receiver operating characteristic curve (AUROC) for each 28-day interval.

**Table 2:** Baseline characteristics

	Study cohort (*n* = 253)
Age, mean (SD)	63.5 (10.2)
Sex, female (%)	48 (19.0)
Body mass index, mean (SD)	28.1 (6.1)
Medical history, *n* (%)	
Secondary prevention ICD indication	176 (69.6)
Diabetes mellitus	42 (16.6)
Atrial fibrillation	87 (34.4)
Percutaneous coronary intervention	89 (35.2)
Coronary artery bypass grafting	49 (19.4)
Hypertension	130 (51.4)
Heart failure (HFrEF)	129 (51.0)
Heart failure (HFpEF)	21 (8.3)
Cardiac resynchronization therapy	39 (15.4)
Ischaemic heart disease	129 (51.0)
Hypertrophic cardiomyopathy	10 (4.0)
Long-QT syndrome	1 (0.4)
ARVC	11 (4.3)
Brugada syndrome	3 (1.2)
Medication, *n* (%)	
ACEi	103 (40.7)
ARB	59 (23.3)
Loop diuretics	84 (33.2)
β-Blocker	208 (82.2)
Antiarrhythmic medication	
Class I	9 (3.6)
Class II	2 (0.8)
Class III	43 (17.0)
NOAC	66 (26.1)
Vitamin K antagonist	34 (13.4)

ACEi, angiotensin-converting enzyme inhibitor; ARB, angiotensin II receptor blocker; ARVC, arrhythmogenic right ventricular cardiomyopathy; HFpEF, heart failure with preserved ejection fraction; HFrEF, heart failure with reduced ejection fraction; ICD, implantable cardioverter defibrillator; NOAC, non-vitamin K anticoagulant; SD, standard deviation.

All data handling and processing were performed using Python (version 3.6.7) with the scikit-survival library (version 0.22.1) and R (version 4.3.2). A *P*-value of <0.05 was considered statistically significant.

## Results

**Figure 1:**
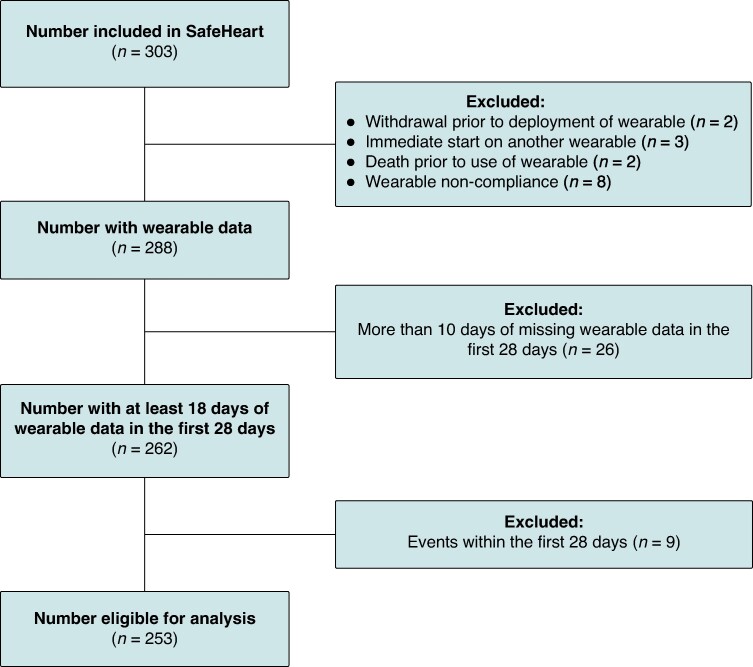
Flowchart of participant inclusions. Flowchart of participants eligible for analysis.

Out of the 303 participants included in the SafeHeart study, a total of 253 were eligible for this analysis. Reasons for exclusion are seen in *Figure 1*. The mean age of eligible participants was 63.5 (10.2), and 48 (19.0%) were female (*Table 2*). Participants who did not meet the minimum adherence criterion at study baseline were older compared to the study cohort (63.5 (±10.2) vs. 67 (±7.5), *P* = 0.032), as seen in Supplementary material online, *Table S1*. During follow-up, 40 participants received appropriate ICD therapy, of which 32 were appropriate ATP, 5 were appropriate shock, and 3 were ATP and shock. Five participants received inappropriate ICD therapy (2.0%) of whom two received inappropriate ATP only and three received inappropriate shock, and six participants died. The cause of death was cardiac in two participants and non-cardiac in four participants. *Table 3* shows the behavioural measurements of the patient cohort. Participants had on average 23.2 days (±4.3) of wearable data collected. Participants were on average inactive for 13.6 h/day [13.2–14.5], time spent in MVPA was 3.1 h [2.7–3.8], median total sleep duration was 6.4 h [6.2–7.0], and the median sleep efficiency was 66.5% [64.8–69.3] per 24 h. Results showed no statistically significant difference in behavioural measures between participants included in the study and those that were excluded due to receiving an appropriate ICD therapy during the blanking period.

**Table 3:** Behavioural measures in the overall population, and those with and without appropriate ICD therapy

Median [IQR]	Total cohort (*n* = 253)	Appropriate therapy (*n* = 40)	No appropriate therapy (*n* = 213)	*P*-value
Activity				
Active events (count)	261.5 [248.3–283.1]	273.9 [249.6–293.7]	261.0 [248.0–278.0]	0.081
Inactive duration (hours)	13.6 [13.2–14.5]	13.9 [13.1–15.8]	13.6 [13.2–14.3]	0.290
MVPA duration (hours)	3.1 [2.7–3.8]	3.2 [2.8–4.1]	3.1 [2.6–3.8]	0.368
M6 intensity (m*g*)	0.3 [0.3–0.3]	0.3 [0.3–0.3]	0.3 [0.3–0.3]	0.141
Average cadence (steps/min)	52.1 [51.0–52.8]	51.9 [50.4–52.7]	52.1 [51.0–52.9]	0.287
Peak cadence (steps/min)	98.2 [92.9–104.5]	102.5 [97.5–109.6]	97.2 [92.7–103.8]	0.001
Activity volume (m*g*·s)	2820.1 [2492.9–3216.0]	2888.6 [2635.0–3643.0]	2813.4 [2484.0–3199.6]	0.248
Sleep				
Total sleep duration (hours)	6.4 [6.2–7.0]	6.7 [6.2–7.3]	6.4 [6.1–6.9]	0.035
Sleep efficiency (%)	66.5 [64.8–69.3]	67.3 [64.5–70.3]	66.5 [64.8–69.1]	0.622
Wake after sleep onset events (count)	28.5 [27.2–31.7]	28.8 [27.2–33.1]	28.5 [27.2–31.7]	0.848
Number of sleep events (count)	127.2 [120.1–138.2]	128.5 [121.1–138.9]	127.2 [120.1–138.0]	0.955

*g*, units of gravity acceleration; *g*·s, units of gravity acceleration seconds; MVPA, moderate to vigorous physical activity; M6 intensity, the intensity of the most active 6 min within a 24 h period; peak cadence, the 95th percentile of mean cadence.

### Associations between physical behaviour and the outcome

**Figure 2:**
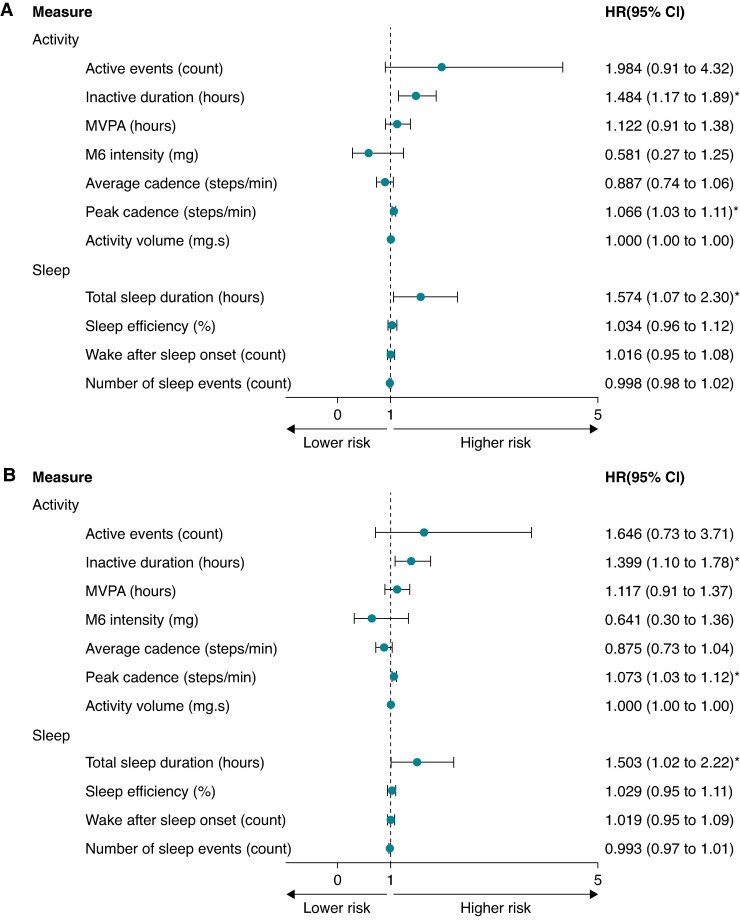
Forest plot showing the hazard ratio (HR) and 95% confidence intervals (CIs) for physical behaviours and appropriate ICD therapy, (*A*) unadjusted and (*B*) adjusted for clinical confounders. (*B*) Adjusted for age, sex, heart failure diagnosis, ischaemic heart disease, device type (CRT-D vs. ICD), and prevention type (primary vs. secondary). CRT-D, cardiac resynchronization therapy; *g*, units of gravity acceleration; *g*·s, units of gravity acceleration seconds; ICD, implantable cardioverter defibrillator; MVPA, moderate to vigorous physical activity; M6 intensity, the intensity of the most active 6 min within a 24 h period; peak cadence, the 95th percentile of mean cadence; *, *P* <0.05.

As shown in *Table 4*, the rate of appropriate ICD therapy per 100 person-years was 19.13 (95% 13.66–26.05) for the overall population. The cumulative incidence for appropriate ICD therapy was 15.0% (95% CI 10.6–19.4%) during follow-up. *Figure 2A* shows the unadjusted HRs for the behavioural measures. Inactive duration (HR 1.48, 95% CI 1.17–1.89), peak walking cadence (HR 1.07, 95% CI 1.03–1.11), and total sleep duration (HR 1.57, 95% CI 1.07–2.30) were all positively associated with risk of the outcome. These associations remained in the adjusted model (*Figure 2B*). No other physical behaviour measures exhibited any statistically significant associations with appropriate ICD therapy. In the sensitivity analysis, we found no significant associations between behavioural measures and either inappropriate ICD therapy or appropriate shock-only treatment.

**Table 4:** Event rates per 100 person-years for appropriate ICD therapy in the cohort overall and the below- vs. above-median inactive duration, peak cadence, and sleep duration groups

	Overall cohort (95% CI)	Inactive duration (95% CI)			Peak cadence (95% CI)			Total sleep duration (95% CI)		
		Below	Above	*P*-value^a^	Below	Above	*P*-value^a^	Below	Above	*P*-value^a^
Event rates per 100 person-years	19.13 [13.66–26.05]	17.0 [10.1–26.8]	21.4 [13.4–32.3]	0.79	9.0 [4.3–16.6]	30.6 [20.6–43.6]	<0.001	12.9 [7.0–21.6]	25.9 [16.9–38.0]	0.045

CRT-D, cardiac resynchronization therapy; ICD, implantable cardioverter defibrillator; CI, confidence interval.

^a^Cox proportional hazard models adjusted for age, sex, heart failure diagnosis, ischaemic heart disease, device type (CRT-D vs. ICD), and prevention type (primary vs. secondary).

**Figure 3:**
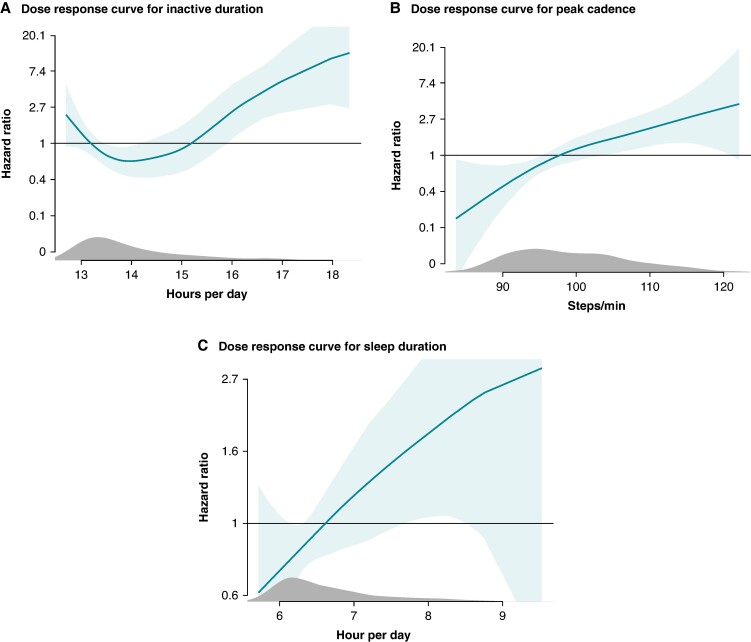
Dose–response relationship between appropriate ICD therapy and (*A*) inactive duration per day, (*B*) peak cadence, (*C*) total sleep duration. Dose–response curves showing the relationship between the dose of physical behaviours and the association to appropriate ICD therapy.

We explored the dose–response relationship between the statistically significant behavioural measures and appropriate ICD therapy (*Figure 3*). The association between the time spent inactive and appropriate ICD therapy was U-shaped (*P* = 0.007). Daily inactive duration of >16 h was associated with the outcome [HR 2.45 (95% CI 1.16–5.17)], which increased to a HR of 10.81 (95% CI 3.04–38.51) for >18 h of inactivity per day compared to periods with less inactivity. The dose–response relationship between peak walking cadence, sleep duration, and appropriate ICD therapy was linear.

### Survival analyses

**Figure 4:**
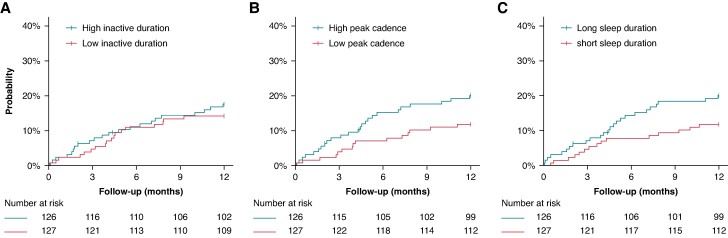
Survival curves for a below-median and above-median (*A*) inactive duration, (*B*) peak cadence, (*C*) sleep duration, and the risk of appropriate ICD therapy. Survival curves censored for death.

The cohort was split into two groups, each comprising participants with above-median or below-median inactive duration, peak walking cadence, and sleep duration. The time-to-event curves are displayed in *Figure 4A–C*. In participants with high peak walking cadence, the cumulative incidence of appropriate ICD therapy during follow-up was 22.2% (95% CI 15.0–29.5), compared to 7.9% (95% CI 3.2–13.0) for low peak walking cadence (*P* < 0.001). No differences in cumulative incidences were observed in above vs. below the median inactive duration and total sleep duration. The three behavioural measures were further stratified into quartiles as displayed in Supplementary material online, *Figure S1A–C*. The lack of significance for inactive duration persisted despite stratification, likely due to the U-shaped dose–response relationship with appropriate ICD therapy.

### Prediction model

**Figure 5:**
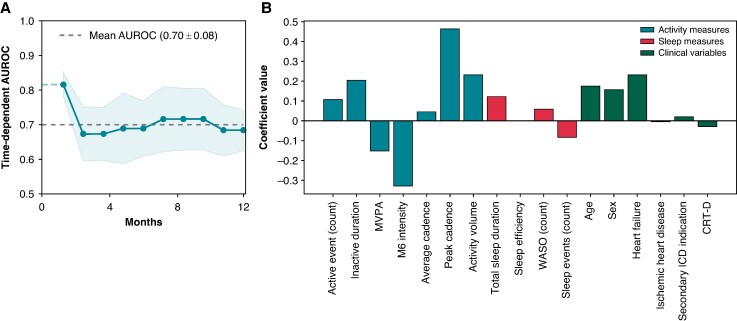
The (*A*) predictive performance of the machine learning prediction model and (*B*) feature importances of modelled variables. AUC, area under the curve; AUROC, area under the receiver operating characteristic curve; CRT-D, cardiac resynchronization therapy defibrillator; MVPA, moderate-vigorous physical activity; M6 intensity, the intensity of the most active 6 min within a 24 h period 6 min walking test; WASO, wake after sleep onset.

The machine learning prediction model incorporating both baseline behavioural measures and clinical variables was evaluated using five-fold cross-validation. The model achieved an average AUROC of 0.70 ± 0.03 for predicting appropriate ICD therapy (*Figure 5A*). Notably, the model exhibited its highest accuracy within the initial months following the collection of wearable data. *Figure 5B* illustrates the feature importances of the model inputs, highlighting activity-related features as having the greatest impact on model predictions. Specifically, M6 intensity and peak walking cadence emerged as the most important features with opposite direction effects.

## Discussion

In summary, we observed associations between appropriate ICD therapy and daily inactive duration, peak walking cadence, and sleep duration in a cohort of 253 participants wearing a wrist-based accelerometer. These associations persisted in multivariable regression analysis accounting for patient characteristics, which suggests that these physical behavioural factors may serve as independent risk factors for appropriate ICD therapy. Dose–response analyses indicated a non-linear relationship between daily inactive duration and the risk of appropriate ICD therapy, where risk of the event increased steeply from 16 h of inactivity and beyond. Conversely, the relationships for peak walking cadence and sleep duration with the outcome were linear, with the risk gradually increasing with larger doses. Survival prediction through behavioural measures and clinical patient characteristics reached good predictive performance (AUROC 0.70 ± 0.03).

This study presents three important novelties. First, while previous studies only examined activity or sleep characteristics in isolation, we were able to assess the entire 24 h activity-sleep cycles, and evaluate their associations to the risk of appropriate ICD therapy.^8,14–16^ Considering that physical behaviours are interconnected and can influence each other in various ways, we used a 24 h framework to achieve a comprehensive understanding of patterns and trends in an individual’s behavioural profile. Second, we aimed to provide an accurate reflection of an individual’s behaviour by using an extended monitoring period of 28 days, a high threshold for valid days (at least 22 h of wear time daily), thus including all types of awake activities and sleep. Third, this is the first study to prospectively assess associations between physical behaviour and the risk of appropriate ICD therapy in a large patient cohort.

### Measuring of activity

Within the ICD itself is an embedded accelerometer. Device embedded accelerometers usually focus on basic activity detection through unidirectional rather than complex multi-directional activity tracking as seen in consumer and research graded wearables.^17^ Through vendor specific algorithms information is converted and is upon device interrogation presented as the percentage of time spent active per every 24 h period.^18,19^ Contrarily, raw data collected from wearable activity trackers have the potential of being universally processed to generate specific metrics, including performance during the most active part of the day, variability measures, time spent at various activity intensities, sedentary periods, rest-activity patterns, and importantly so, also sleep behaviour to ensure round-the-clock behaviour.^17^ With regard to comparative studies, a moderate correlation has been found between activity measured through device embedded vs. external wearable accelerometry, generally underestimating the total time spent active when measuring activity through the ICD.^17,19^ We thus aimed to uncover additional non-invasive, prognostic digital biomarkers for ICD therapy risk, by utilizing a more comprehensive set of behavioural measures.

### Inactive duration and effects on ICD therapy

Sedentary behaviour, characterized by prolonged sitting or reclining with little to no physical activity, has been associated with an increased risk of cardiovascular disease.^3–5,20,21^ In this cohort of patients implanted with an ICD, a higher level of daily inactive duration was associated with increased risk of appropriate ICD therapy. The level of inactivity is known to be affected by various factors, including age and the presence of comorbidities such as heart failure.^21–23^ Interestingly, in adjusted regression analyses that accounted for clinical patient characteristics including the abovementioned, the association between daily inactivity levels and risk of appropriate ICD therapy remained, particularly for levels exceeding 16 h per day. This observations was in line with previous analyses on data of 98 893 UK Biobank participants, which observed an inverse relationship between the risk of cardiac arrest and total physical activity.^3^ In addition, the U-shaped dose–response pattern found in this paper displays a complex relationship where rather than a constant change in risk of appropriate ICD therapy across all levels of inactivity, particularly prolonged periods of inactivity increased the risk of ICD therapy. Additionally, with a U-shaped relationship pattern, the shortest durations of inactivity were also associated with increased risk of the outcome. This pattern may be an effect of fragmentation, indicating that defined periods of restorative rest between active periods are needed. Similarly, one meta-analysis of self-reported sedentary time and cardiovascular disease found a cut-off for increased risk of the outcome of 10 h.^24^ Another meta-analysis of step counts measured by accelerometers, all-cause mortality, and cardiovascular events showed the greatest risk with step counts in the first quantile, compared to the higher step count level of the third quantile.^25^ Understanding this nonlinearity is crucial for developing targeted interventions in individuals with an ICD, to mitigate the risks associated with inactivity.^26^ Lastly, physical inactivity leads to physiological and metabolic changes that particularly impact cardiometabolic health,^27,28^ which is why inactive behaviour may in fact be a proxy for overall health status.^29^

### Activity and effects on ICD therapy

An adequate level of daily moderate to vigorous intensity activity confers numerous health benefits by reducing the burden of cardiovascular disease and mortality, thereby promoting overall health.^4,5,20,30^ Despite these health benefits of PA, there is the so called ‘paradox of physical activity’, where the risk of sudden cardiac arrest is increased during or shortly after vigorous PA.^31–33^ Our findings highlighted that a higher peak walking cadence increased the risk of ventricular arrhythmia. This could be extrapolated to high-bursts of activity putting a greater burden on the heart or leading to transient cardiac injury.^34–36^ In our study, the risk associated with these higher peak walking cadences was not accompanied by increased activity volume or higher intensities for the most active 6 min of the day, suggesting that these bursts were very short and non-incremental to normal daily activity levels. With regard to the transient window of episodes of high-intensity physical activity, one study showed that if you are more sedentary, bursts of activity increased the risk of ventricular arrhythmia.^37^ Contrary, habitually higher activity levels may be protective of this same risk of ventricular arrhythmia.^37^ Moreover studies looking at mortality have shown that activity until a certain level is beneficial, after which the effects seem to wear off in dose–response analyses.^14,38^ Lastly, evenly distributed activity over the week vs. bursts of activity had similar positive effects on risk of heart failure and myocardial infarction.^39^

### Sleep behaviours

The inclusion of sleep measures derived from wearable accelerometry varies significantly across studies, as many studies applied wear time criteria for valid days that excluded hours spent sleeping.^40^ Previous studies that did include sleep behaviours have primarily relied either on self-reporting though questionnaires, or on use of short accelerometry-assessed monitoring intervals of 7 days.^41–44^ Our findings demonstrated that total sleep duration was associated with appropriate ICD therapy, however, it is unclear what the precise mechanism is for the association between sleep behaviour and arrhythmia risk. In prior studies, disturbed rest-activity cycles have been associated with increased risk of heart failure progression, atrial fibrillation, and ventricular arrhythmia.^42–44^ Moreover, two studies using accelerometry-assessed sleep behaviour measured over a 7-day period, showed that patients with heart failure had disturbed rest-activity patterns compared to healthy controls.^45^ In addition, a relationship between the risk of developing cardiovascular disease and sleep onset timing has been previously reported.^41^ Noteworthy is that our results did not show a separation in survival curves between those that had an above and below median sleep duration until after 4 months of study participation. This may be suggestive of a cumulative effect of sleep duration on cardiovascular health, where the impact of different sleep durations might not be immediately apparent. Over time, however, prolonged exposure to short or long sleep durations could lead to significant physiological changes.^46^ Alternatively, longer sleep durations may in itself be a sign of worsening cardiac health, relatable to the increase in the associated risk of appropriate ICD therapy seen in our study.^47^ Moreover, the fact that longer durations of sleep show an association with increased risk of ICD therapy but wake after sleep onset (WASO) did not could involve different ways these sleep metrics may impact cardiovascular health.

Extended total sleep duration might be indicative of underlying health issues such as poor sleep quality, excessive daytime sleepiness, or comorbid conditions that are linked to an increased risk of arrhythmias or other cardiovascular problems.^47^ In contrast, WASO, which measures the time spent awake after initially falling asleep, might reflect less direct or less consistent impacts on cardiovascular health. Thus, while long total sleep duration could signal significant health problems or disruptions in sleep architecture that contribute to increased ICD therapy risk, WASO may not capture these effects as clearly or may be less directly related to the risk of ICD therapy. Additionally, the variability in WASO among individuals, study power or measurement challenges could contribute to its lack of statistical significance in this context, as a previous community study found an increased risk of cardiovascular disease when the total WASO was longer than 78 min.^48^ Our results show promise for more granular, accelerometry-assessed sleep measures and ICD therapy risk. It is therefore crucial to further explore this role of sleep behaviour on the risk of cardiac arrhythmias.

### Prediction model

The prediction model employed in this study demonstrated the highest accuracy in the months immediately following data collection. This is likely due to the temporality of the input variables, whose predictive power diminish as the time gap increases. Furthermore, changes to activity measures or clinical parameters may occur over time that interfere with the results of the initial prediction.

### Clinical implications

The use of a random 28-day window for data collection offers flexibility in measuring behavioural trends independently of the immediate post-ICD implantation period. However, the predictive model indicates that predictions are strongest within a limited time following this data collection, whereafter renewed measuring may be indicated. Despite our results being early, they suggest that behavioural activity and sleep patterns hold significant predictive value. Incorporating these factors with other machine learning methods could further enhance the prediction of ICD therapy needs. Health professionals should be particularly vigilant if total sleep duration or inactivity exceeds 16 h, as these may be early indicators of health deterioration.

### Limitations

There are several limitations to acknowledge. First, due to the observational nature of this study, there is a risk of residual confounding that may have affected the results. Second, selection bias towards including participants with greater health capacity cannot be excluded as our cohort demanded active consent from participants and acceptance to use a wearable with high adherence in the 28 days used for study. Third, the sample size in our study is relatively small which inherently affects the statistical power of our findings, particularly that of less common outcomes such as inappropriate ICD therapy and shock-only therapy. This emphasizes the need for further testing in larger prospective cohorts. Fourth, the present ICD cohort is not necessarily generalizable to other patient cohorts, since it is important to note that 81% of our participants were male, and results may not be representative of female ICD carriers. Fifth, we used activity data from the first month as baseline, which means that potential changes in activity over the follow-up period were not accounted for. Consequently, if participants who were inactive during the initial 28 days but later improved their activity levels during follow-up received appropriate therapy, the analysis might overestimate the true extent of inactivity. Sixth, the inclusion criteria specifically targeted ICD-participants at elevated risk of ICD therapy, which may limit the generalizability of the results to a broader ICD population. Lastly, due to the observational nature of the study, the settings of the ICD were not harmonized across participants. Variations in ICD settings and medical therapy inherently impact each patient’s likelihood of receiving appropriate or inappropriate ICD therapy. Consequently, it cannot be rejected that any individual programming may have affected the frequency of appropriate therapies, especially in regard to ATP therapy.

## Conclusion

In this study, we demonstrated that 24 h cycles of activity and sleep behaviours, collected over 28 days, are independently associated with the risk of later appropriate ICD therapy. Inactivity exceeding 16 h was strongly associated with appropriate ICD therapy in a dose–response manner, while the associations between increased peak walking cadence and longer sleep duration were linear. This is the first study to demonstrate the predictive capacity of behavioural measurements obtained from a wearable device for appropriate ICD therapy. Larger studies are warranted to confirm these findings.

## Supplementary Material

euae241_Supplementary_Data

## Data Availability

The data underlying this article will be shared on reasonable request to the corresponding author.
