# Supplementary material for: Relationship between activity and sleep, as measured through a wearable accelerometer, and appropriate cardioverter defibrillator interventions: a prospective SafeHeart substudy
Source: Europace. 2024 Sep 20;26(10):euae241. doi: 10.1093/europace/euae241 (PMC11481428; doi:10.1093/europace/euae241)

**Supplementary material**

**Supplementary Table S1**

|  | | | **Study cohort (n=253)** | **Excluded subjects (n=26)** | ***p*** |
| --- | --- | --- | --- | --- | --- |
|  | | |  |  |  |
| Age, mean (SD) | | | 63.5 (10.2) | 67.0 (7.5) | 0.032 |
| Sex, female (%) | | | 48 (19.0) | 4 (15.4) | 0.796 |
| Body mass index, mean (SD) | | | 28.1 (6.1) | 27.4 (4.1) | 0.455 |
| **Medical history, n (%)** | | | |  |  |
|  | Secondary prevention ICD indication | | 176 (69.6) | 14 (53.8) | 0.157 |
|  | Diabetes mellitus | | 42 (16.6) | 3 (11.5) | 0.779 |
|  | Atrial fibrillation | | 87 (34.4) | 13 (50.0) | 0.172 |
|  | Percutaneous coronary intervention | | 89 (35.2) | 7 (26.9) | 0.531 |
|  | Coronary artery bypass grafting | | 49 (19.4) | 3 (11.5) | 0.434 |
|  | Hypertension | | 130 (51.4) | 14 (53.8) | 0.973 |
|  | Heart failure (HFrEF) | | 129 (51.0) | 18 (69.2) | 0.117 |
|  | Heart failure (HFpEF) | | 21 (8.3) | 2 (7.7) | 1.000 |
|  | Cardiac resynchronization therapy | | 39 (15.4) | 6 (23.1) | 0.397 |
|  | Ischemic heart disease | | 129 (51.0) | 9 (34.6) | 0.166 |
|  | Hypertrophic cardiomyopathy | | 10 (4.0) | 1 (3.8) | 1.000 |
|  | Long-QT syndrome | | 1 (0.4) | 0 (0) | 1.000 |
|  | ARVC | | 11 (4.3) | 4 (15.4) | 0.040 |
|  | Brugada syndrome | | 3 (1.2) | 0 (0) | 1.000 |
| **Medication, n (%)** | | | |  |  |
|  | ACEi | | 103 (40.7) | 10 (38.5) | 0.990 |
|  | ARB | | 59 (23.3) | 9 (34.6) | 0.299 |
|  | Loop diuretics | | 84 (33.2) | 13 (50.0) | 0.135 |
|  | β-blocker | | 208 (82.2) | 5 (19.2) | 0.792 |
|  | Antiarrhythmic medication | |  |  |  |
|  |  | Class I | 9 (3.6) | 2 (7.7) | 0.273 |
|  |  | Class II | 2 (0.8) | 1 (3.8) | 0.255 |
|  |  | Class III | 43 (17.0) | 5 (19.2) | 0.786 |
|  | NOAC | | 66 (26.1) | 11 (42.3) | 0.126 |
|  | Vitamin K antagonist | | 34 (13.4) | 5 (19.2) | 0.382 |
|  | **Abbreviations**: ACEi, Angiotensin-converting enzyme inhibitor; ARB, angiotensin II receptor blocker; ARVC, arrhythmogenic right ventricular cardiomyopathy; HFpEF, heart failure with preserved ejection fraction; HFrEF, heart failure with reduced ejection fraction; ICD, implantable cardioverter defibrillator; NOAC Non-vitamin K anticoagulant; SD, standard deviation | | | | |

**Supplementary Figure S1**

Survival curves for quartiles of (a) inactive duration, (b) peak cadence, (c) sleep duration and the risk of appropriate ICD-therapy


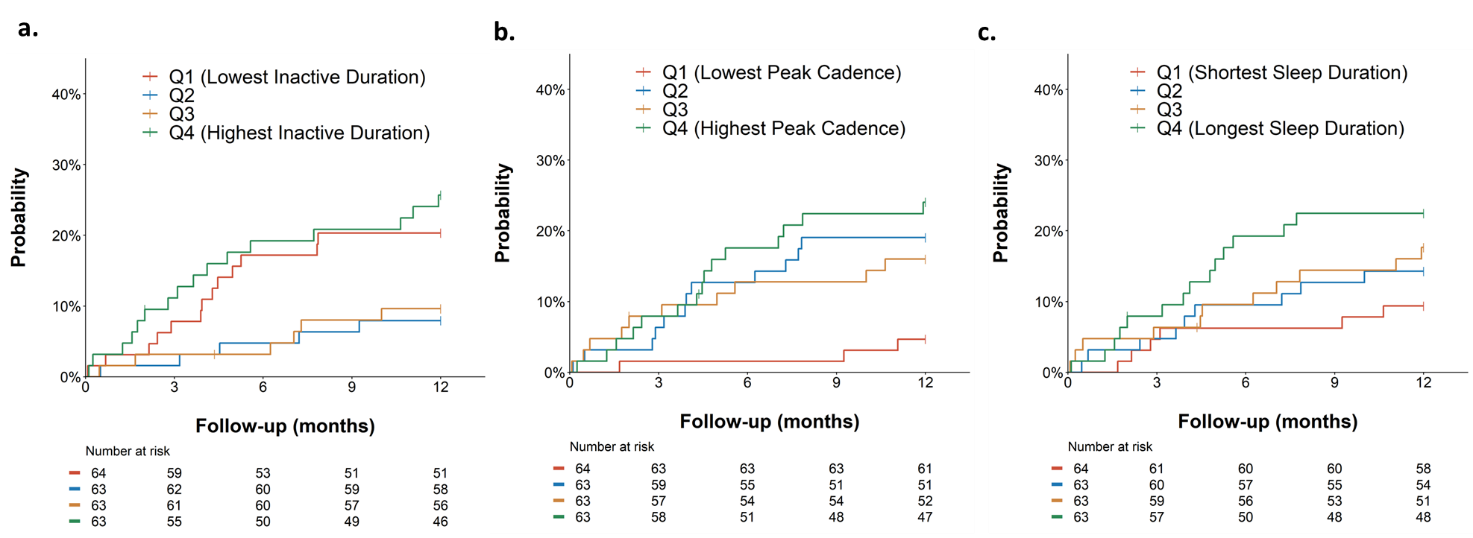

Supplement: euae241_Supplementary_Data [file euae241_supplementary_data.docx]
